# Mammotome-assisted endoscopic breast-conserving surgery: a novel technique for early-stage breast cancer

**DOI:** 10.1186/1477-7819-12-99

**Published:** 2014-04-18

**Authors:** Yan Xu, Jia Ming, Yan Zhou, Xiaowei Qi, Linjun Fan, Jun Jiang

**Affiliations:** 1 Breast Disease Center, Southwest Hospital, Third Military Medical University, 400038 Chongqing, People’s Republic of China

**Keywords:** Mammotome, Endoscopic surgery, Early-stage breast cancer

## Abstract

**Background:**

Because of its minimally invasive and highly accurate nature, the use of Mammotome, a vacuum-assisted breast biopsy device has proven beneficial to the treatment of benign breast lesions. Taking advantage of endoscopic and Mammotome techniques together, we utilized the Mammotome device for therapeutic excision of malignant lesions in breast-conserving surgery (BCS).

**Methods:**

Between December 2009 and January 2010, two patients with early breast cancer received Mammotome-assisted endoscopic BCSs. Under ultrasound monitoring, the Mammotome system dissected the surrounding tissue and freed the tumor *en bloc* leaving negative margins; endoscopic axillary lymph node dissection then followed.

**Results:**

The operation time was less than 180 minutes and the mean blood loss was 60 ml. The post-operative pathology report confirmed two patients to have invasive ductal carcinoma, one without axillary lymph nodes metastasis (0/11) and the other with one lymph node metastasis (1/21). No adverse events were noted. During a mean follow-up of 26.5 months, no evidence of recurrence or metastasis was found. The patients were satisfied with the cosmetic results.

**Conclusions:**

Mammotome-assisted endoscopic surgery appears to be a valuable option for early breast cancer. The long-term therapeutic effect remains to be confirmed.

## Background

One of the current standards of approach for early-stage breast cancer is breast-conserving surgery (BCS) [1]. However, clinically, a minority of breast cancer patients with BCS are still dissatisfied with their cosmetic outcome [2,3]. In order to avoid the obvious incision of the breast and obtain a better cosmetic outcome, endoscope-assisted surgery has been adapted in BCS, but the applications are restricted because of the long operation time involved and relatively large blood loss [4].

The most commonly used vacuum-assisted biopsy device, Mammotome (Ethicon Endo-surgery, Inc., Cincinnati, OH, USA), has been successfully employed for excision of benign breast lesions such as fibroadenoma and intraductal papillomas due to its advantages of minimal invasiveness and more precise tissue harvesting [5,6]. However, until now, there have been few reports on its application in resecting malignant breast tumors. In this study, we used a Mammotome-assisted endoscopic technique to excise the early breast cancer, improve the accuracy and completeness of excision and acquire a better appearance in two patients.

## Methods

Between December 2009 and January 2010, two patients underwent Mammotome-assisted endoscopic excision technique of a preoperatively diagnosed invasive breast cancer at our hospital. Table 1 summarizes their characteristics. The two patients were women. On examination, one patient’s tumor was localized in the upper-outer quadrant of the left breast near the axilla (2.1 cm maximum diameter) and one enlarged lymph node which might have been involved was located in the ipsilateral axilla by ultrasonography (clinical stage: T2,N1,M0). The other patient’s tumor was 3.0 cm in maximum diameter and localized in the upper-inner quadrant of the left breast by preoperative ultrasonography (clinical stage: T2,N0,M0). Both patients’ hormone receptor status was estrogen receptor (ER)/progesterone receptor (PR) positive and epidermal growth factor receptor 2 (HER-2) negative. After four cycles of neoadjuvant chemotherapy with the DE regimen (docetaxel 75 mg/m^2^ and epirubicin 80 mg/m^2^ on day 1, every 2 weeks), ultrasound examinations showed that the tumors were reduced to 0.90 cm and 0.96 cm respectively in the maximum diameter and partial remission was achieved according to tumor response criteria (Response Evaluation Criteria in Solid Tumors, RECIST) [7]. All patients underwent Mammotome-assisted endoscopic breast-conserving surgery. The surgical procedures are described below.

**Table 1:** Clinical characteristics of the two patients before surgery

**Patient number**	**Age/sex**	**Tumor size (cm)**	**TNM**	**Hormone status**	**HER-2**	**NACT regimen**	**Tumor size (cm) after NACT**
1	57/F	2.1	T2N1M0	+	-	Docetaxel + epirubicin	0.9
2	41/F	3.0	T2N0M0	+	-	Docetaxel + epirubicin	0.96

NACT: Neoadjuvant chemotherapy.

### Consent

Written informed consent was obtained from all the patients for publication of this report and any accompanying images.

### Surgical procedures

**Figure 1:**
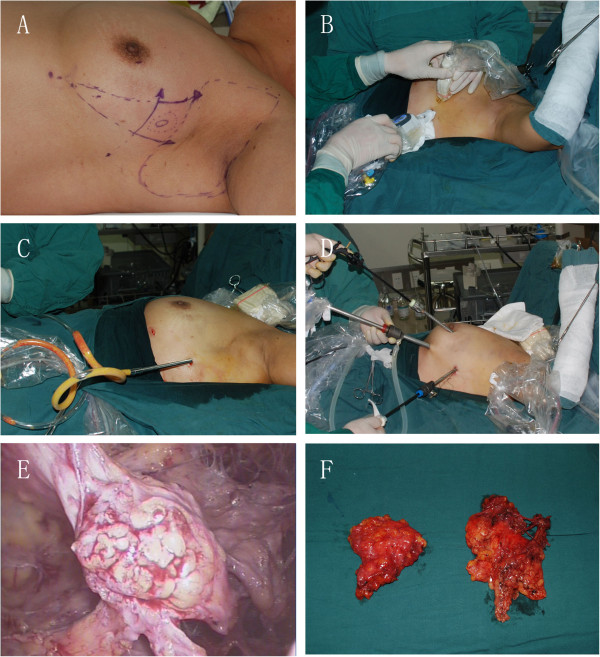
**The detailed process of the Mammotome-assisted endoscopic breast-conserving surgery for case 1. (A)** The edges of the tumor to be cut and the axillary border were marked. **(B)** The tissues in inferior margin of tumor were removed. **(C)** The fat was aspirated. **(D)** Endoscope and operating instruments were inserted into the subcutaneous space. **(E)** Tumor’s endoscopic image. **(F)** Resected specimens.

The patient was placed in the supine position, with the arm abducted to 90° after the induction of general anesthesia. The cutting edges were marked at a distance of 5 mm from the tumor, and at the lower exterior margin of the breast, the intersection points of the nipple level line and anterior border of the latissimus dorsi muscle were marked as the puncture sites for the Mammotome (Figure 1A). The lipolysis solution (250 ml of saline, 250 ml of distilled water, 1 mg epinephrine and 400 mg lidocaine) was injected into the subcutaneous space above the lesion, retromammary spaces and axillary fossa. A 5-mm incision was made in the skin at nipple level Mammotome puncture site and then an 8G probe was inserted into the posterior of the tumor via the retromammary spaces under ultrasonic guidance. The probe aperture was turned facing up towards the tumor and after rotation of the probe, the first 12 biopsies were taken and the deep, superior, inferior and superficial margins of the tumor were removed. Then, another 5-mm incision was made at the lower exterior margin of breast as the second puncture site, the Mammotome probe was inserted into tumor area, a further seven biopsies were taken and the medial and lateral margins of the tumor were resected (Figure 1B). All of the tissue margins were shown to be negative by intra-operative frozen section analysis (FSA). Sufficient liposuction was performed in the axillary, subcutaneous and retromammary spaces of the breast via the two Mammotome puncture sites using a metal aspiration tube with a side aperture (a suction tip used for uterine curettage); (Figure 1C). Another 5-mm incision was made at the upper exterior margin of the breast areola, with two Mammotome puncture sites as trocar sites. CO_2_ pressure was infused into the axilla to approximately 8 mmHg and then a 30-degree 10-mm endoscope was inserted into the subcutaneous space through the trocar sleeve. The trocar sleeve was placed in the lower exterior margin incision of breast, and the operating instruments were inserted into the other two trocars (Figure 1D). Under endoscopy, we found the tumor had been almost dissected (Figure 1E). Fibrous tissues connected with the tumor were removed and then levels I and II axillary lymph nodes were dissected endoscopically. The endoscopic dissection method of axillary lymph nodes was described previously [8]. The dissected tumor and axillary specimen (Figure 1F) were extracted through the axillary incision. A suction drainage tube was inserted into the axillary space through the inferior trocar hole.

**Figure 2:**
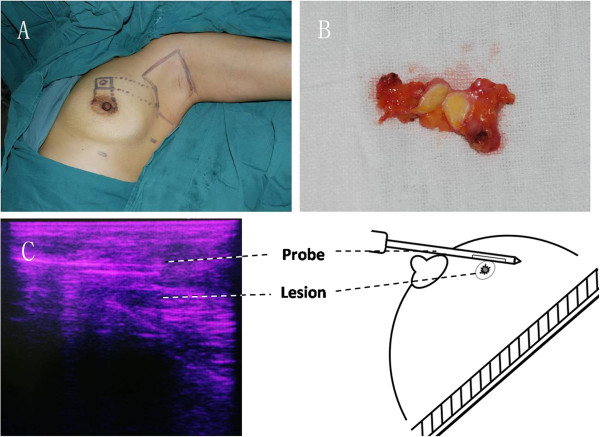
**The Mammotome-assisted endoscopic breast-conserving surgery used for case 2. (A)** Cutting edges of tumor and axilla were marked. **(B)** Dissected tumor and the surrounding normal tissues. **(C)** Ultrasound assessment for the Mammotome procedure and schematic representation of Mammotome excision.

The anesthesia and positioning of patient 2 was same as patient 1. The cutting edges were marked at a distance of 5 mm from the tumor. The intersection points of the cutting edges extension line with the upper margin of areola and the upper exterior margin of the breast were marked as skin puncture sites of the Mammotome (Figure 2A). After injecting lipolysis solution, normal tissues on the marked cutting edges around the tumor were excised by the Mammotome device under ultrasound guidance. The resected tissue was obtained for intra-operative FSA and was shown to have negative margins. A 3-cm peri-areolar incision was made, and the subcutaneous space from the incision to the cavity around the tumor was freed. The small amount of mammary gland tissue remaining connected around the lesion was removed. The tumor was dissected and removed via the incision (Figure 2B, and C). A 10-mm incision was made in the lower exterior margin of breast, and another 5-mm incision was made at the intersection point of anterior border of the latissimus dorsi muscle and the nipple level line. Lipolysis and liposuction of the axillary fossa and endoscopic axillary lymph node dissection were performed as mentioned above.

## Results

The operation time and the blood loss of each patient is shown in Table 2. The pathology report was that the margins (medial, lateral, superior, inferior, superficial, and deep) of each breast mass were uninvolved with tumor. Two cycles of chemotherapy with DE regimen were administered post-operatively. Post-operative radiation therapy was administered, followed by endocrine therapy. The post-operative pathology report confirmed the two patients as having invasive ductal carcinoma, but no metastases were demonstrated in the dissected axillary lymph nodes (0/11) of one, and one lymph node metastasis (1/21) was demonstrated in the other.

**Table 2:** Clinical outcomes for patients after surgery

**Patient number**	**Operation time (minutes)**	**Lymph node status**	**Blood loss (ml)**	**Drainage time (days)**	**Drainage volume (ml)**	**Chemotherapy regimen**	**Radiation therapy**	**Follow-up (months)**	**Complications**
1	175	0/11	70	10	387	Docetaxel + epirubicin	Yes	27	None
2	150	1/21	50	7	227	Docetaxel + epirubicin	Yes	26	None

**Figure 3:**
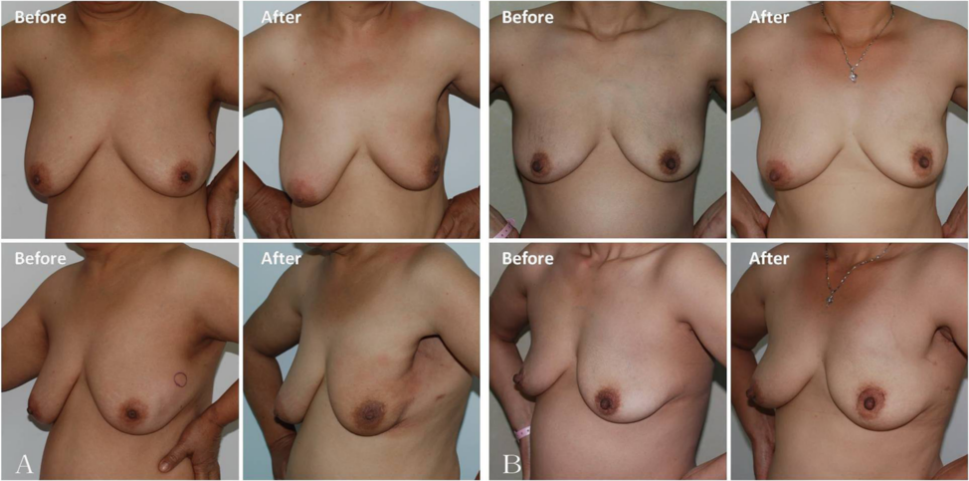
**Pre- and post-operative images of case 1 and case 2. (A)** Case 1, 18 months after surgery. **(B)** Case 2, 12 months after surgery.

The patients received follow-up evaluation every six months. They remained well and disease-free for over 26 months after the operation. No evidence of recurrence or metastasis was found. No adverse events associated with endoscopic axillary lymph node dissection had been observed at the time of writing. Furthermore, two patients were very satisfied with the cosmetic results (Figure 3A and B for patients 1 and 2, respectively).

## Discussion

Endoscopic surgery has been used in the treatment of breast cancer for over a decade [9-12]. As a minimally invasive technique, endoscopic surgery has prominent advantages over the traditional open approach. Many clinical trials have demonstrated that endoscope-assisted BCS is safe and feasible for early-stage breast cancer [13,14]. Ultrasound-guided Mammotome biopsy is widely recognized as an accurate and valuable option for the diagnosis of breast lesions [15], and this procedure has recently been highly recommended and increasingly applied in the treatment of benign breast lesions [5,6]. However, it has rarely been recommended for malignant breast tumor removal in accordance with the *en bloc* resection principle of malignant tumors.

Employing the advantages of the Mammotome, we expected that a vacuum-assisted biopsy system could be used to remove non-cancerous surrounding tissues before performing complete excision of a small size tumor rather than directly cutting the tumor into pieces. In our studies, we combined the Mammotome and endoscopic techniques in BCS and used the Mammotome system for therapeutic excision of malignant lesions. Under ultrasound guidance, the Mammotome system facilitated the removal of the cutting edges, approximately 4 mm in thickness, around the tumor, and each specimen was sent for intra-operative FSA. Using this technique, after the tumor had been almost completely separated, we could conserve the normal breast tissue adjacent to the tumor as much as possible. If the distance between tumor and nipple was < 3 cm, the residual breast tissues contiguous to the tumor could be excised through a peri-areolar incision to perform wide local excision surgery. If the tumor-nipple distance was > 3 cm, the tumor dissection could be performed via the endoscopic approach.

Endoscopic axillary lymph node dissection was performed with three trocars individually located at the lower exterior margin of the breast, the upper exterior margin of the areola, intersection points of the nipple level line and the anterior border of the latissimus dorsi muscle (some of these trocar sites could also serve as Mammotome puncture sites). The axillary specimens were extracted from the axillary incision, and the dissected tumor was extracted from the axillary or the peri-areolar incision.

A number of researchers have limited their clinic trials of endoscopic breast cancer surgery to T1 and small T2 tumors [12,16]. Based on our practice, the size and location of the tumor and the tumor-nipple distance should be assessed carefully in Mammotome-assisted endoscopic BCS, because oncologic safety and the convenience of the operation should be given full consideration [17]. The maximum tumor diameter of both patients presented herein was reduced to <1 cm after neoadjuvant chemotherapy. The cutting edges of the Mammotome were 5 mm away from the tumor, and all margin tissues were confirmed to be negative by intra-operative FSA. Theoretically, breast cancer patients with tumors <2 cm in size, located >2 cm from the areola and without invasion of the skin and pectoralis major muscle can safely undergo Mammotome-assisted endoscopic BSC.

An important purpose of this surgery is to obtain better cosmetic results. Small incisions were made in a distal and poorly-visible area with access the tumor, which was then removed with the Mammotome and endoscopic surgery technique. In the two patients described herein, we made another skin incision around the areola or in the axillary fossa through which to excise and remove the tumor, and no breast skin incision or obvious scar was detectable at the operation site. These two patients were very satisfied with the post-operative cosmetic results. No recurrence or metastasis had occurred in follow-up for a mean of 26.5 months.

Additionally, endoscopy-assisted techniques for the removal of malignant tumors have been described previously with better cosmetic outcome than the conventional BCS [14]. However, in open surgery or under an endoscope, without real-time ultrasonic guidance, it is difficult to achieve precise excision of tumor margin along the pre-drawn excision line. Moreover, the traction during operation may cause the actual cutting edges to deviate from the pre-drawn lines, which may lead to insufficient dissection or over-dissection. In Mammotome-assisted endoscopic BCS, the surgeon can accurately dissect the tumor margin and free the tumor under real-time ultrasonic guidance, thereby avoiding deviation from actual excision lines from the pre-drawn ones. Although in case 2, the length of the incision may not show significant advantage compared with minimally invasive open surgery, we believe that the length and location of the incisions can be further improved with the accumulation of our surgical experience.

## Conclusions

In conclusion, this is the first time that a combinative use of Mammotome and endoscopic surgery techniques to perform the *en bloc* resection of breast cancer has been reported. We advocate that Mammotome-assisted endoscopic BCS should be a safe and effective technique for minimally invasive tumor excision with a safety margin in early-stage breast cancer. Additionally, this novel surgical approach could also provide a significantly better cosmetic outcome than conventional lumpectomy for well selected patients. Nevertheless, there are some disadvantages to this technique which are as follow: patients with tumor >2 cm are not suitable for this method; the operation time is longer than that of open surgery; and the operation can only be successfully performed by a surgeon experienced in endoscopic and Mammotome techniques. Moreover, the long-term therapeutic effect still requires further confirmation with a large-sample clinical trial and long-term follow-up.

## Abbreviations

BCS: breast-conserving surgery; ER: estrogen receptor; FSA: frozen section analysis; HER-2: epidermal growth factor receptor 2; NACT: neoadjuvant chemotherapy; PR: progesterone receptor; RECIST: Response Evaluation Criteria in Solid Tumors.

## Competing interests

All authors declare that they have no competing interests.

## Authors’ contributions

Study conception and design: LF, JJ; acquisition of data: YX, JM, YZ, XQ; analysis and interpretation of data: YX; drafting of manuscript: YX; critical revision: LF; supervision: JJ. All authors read and approved the final manuscript.

## References

[B1] Veronesi U Cascinelli N Mariani L Greco M Saccozzi R Luini A Aguilar M Marubini E Twenty-year follow-up of a randomized study comparing breast-conserving surgery with radical mastectomy for early breast cancer N Engl J Med 2002 347 1227 1232 10.1056/NEJMoa020989 12393819

[B2] Al-Ghazal SK Blamey RW Cosmetic assessment of breast-conserving surgery for primary breast cancer Breast 1999 8 162 168 10.1054/brst.1999.0017 14731434

[B3] Yang JD Lee JW Kim WW Jung JH Park HY Oncoplastic surgical techniques for personalized breast conserving surgery in breast cancer patient with small to moderate sized breast J Breast Cancer 2011 14 253 261 10.4048/jbc.2011.14.4.253 22323910 PMC3268920

[B4] Noguchi M Roles of endoscopy-assisted approaches in surgical management of breast cancer: present state of the art and outlook for the future Trends Cancer Res 2006 2 85 92

[B5] Iwuagwu O Drew P Vacuum-assisted biopsy device-diagnostic and therapeutic applications in breast surgery Breast 2004 13 483 487 10.1016/j.breast.2004.06.004 15563855

[B6] Maxwell AJ Ultrasound-guided vacuum-assisted excision of breast papillomas: review of 6-years experience Clin Radiol 2009 64 801 806 10.1016/j.crad.2009.04.007 19589419

[B7] Therasse P Arbuck SG Eisenhauer EA Wanders J Kaplan RS Rubinstein L Verweij J Van Glabbeke M van Oosterom AT Christian MC Gwyther SG New guidelines to evaluate the response to treatment in solid tumors. European organization for research and treatment of cancer, national cancer institute of the united states, national cancer institute of Canada J Nati Cancer Inst 2000 92 205 216 10.1093/jnci/92.3.205 10655437

[B8] Fan LJ Jiang J Surgical technique of fully endoscopic modified radical mastectomy for breast cancer Chin J Breast Dis (electronic version, in Chinese) 2010 4 17 26

[B9] Tamaki Y Nakano Y Sekimoto M Sakita I Tomita N Ohue M Komoike Y Miyazaki M Nakayama T Kadota M Monden M Transaxillary endoscopic partial mastectomy for comparatively early-stage breast cancer. An early experience Surg Laparosc Endosc 1998 8 308 312 10.1097/00019509-199808000-00015 9703608

[B10] Tamaki Y Sakita I Miyoshi Y Sekimoto M Takiguchi S Monden M Noguchi S Transareolar endoscopy-assisted partial mastectomy: a preliminary report of six cases Surg Laparosc Endosc Percutan Tech 2001 11 356 362 10.1097/00129689-200112000-00003 11822858

[B11] Langer I Kocher T Guller U Torhorst J Oertli D Harder F Zuber M Long-term outcomes of breast cancer patients after endoscopic axillary lymph node dissection: a prospective analysis of 52 patients Breast Cancer Res Treat 2005 90 85 91 10.1007/s10549-004-3268-6 15770531

[B12] Owaki T Yoshinaka H Ehi K Kijima Y Uenosono Y Shirao K Nakano S Natsugoe S Aikou T Endoscopic quadrantectomy for breast cancer with sentinel lymph node navigation via a small axillary incision Breast 2005 14 57 60 10.1016/j.breast.2004.05.002 15695082

[B13] Leff DR Vashisht R Yongue G Keshtgar M Yang GZ Darzi A Endoscopic breast surgery: where are we now and what might the future hold for video-assisted breast surgery? Breast Cancer Res Treat 2011 125 607 625 10.1007/s10549-010-1258-4 21128113

[B14] Park HS Lee JS Park S Kim SI Park BW The feasibility of endoscopy-assisted breast conservation surgery for patients with early breast cancer J Breast Cancer 2011 14 52 57 10.4048/jbc.2011.14.1.52 21847395 PMC3148518

[B15] Nakano S Sakamoto H Ohtsuka M Mibu A Sakata H Yamamoto M Evaluation and indications of ultrasound-guided vacuum-assisted core needle breast biopsy Breast Cancer 2007 14 292 296 10.2325/jbcs.14.292 17690507

[B16] Ho WS Ying SY Chan AC Endoscopic-assisted subcutaneous mastectomy and axillary dissection with immediate mammary prosthesis reconstruction for early breast cancer Surg Endosc 2002 16 302 306 10.1007/s004640000203 11967683

[B17] Fitzal F Riedl O Mittlbock M Dubsky P Bartsch R Steger G Jakesz R Gnant M Oncologic safety of breast-conserving surgery after tumour downsizing by neoadjuvant therapy: a retrospective single centre cohort study Breast Cancer Res Treat 2011 127 121 128 10.1007/s10549-010-1164-9 20848185

